# Connectivity of Fronto-Temporal Regions in Syntactic Structure Building During Speaking and Listening

**DOI:** 10.1162/nol_a_00154

**Published:** 2024-10-08

**Authors:** Laura Giglio, Daniel Sharoh, Markus Ostarek, Peter Hagoort

**Affiliations:** Max Planck Institute for Psycholinguistics, Nijmegen, The Netherlands; Donders Institute for Brain, Cognition and Behaviour, Nijmegen, The Netherlands

**Keywords:** comprehension, connectivity, fMRI, language, production, syntax

## Abstract

The neural infrastructure for sentence production and comprehension has been found to be mostly shared. The same regions are engaged during speaking and listening, with some differences in how strongly they activate depending on modality. In this study, we investigated how modality affects the connectivity between regions previously found to be involved in syntactic processing across modalities. We determined how constituent size and modality affected the connectivity of the pars triangularis of the left inferior frontal gyrus (LIFG) and of the left posterior temporal lobe (LPTL) with the pars opercularis of the LIFG, the left anterior temporal lobe (LATL), and the rest of the brain. We found that constituent size reliably increased the connectivity across these frontal and temporal ROIs. Connectivity between the two LIFG regions and the LPTL was enhanced as a function of constituent size in both modalities, and it was upregulated in production possibly because of linearization and motor planning in the frontal cortex. The connectivity of both ROIs with the LATL was lower and only enhanced for larger constituent sizes, suggesting a contributing role of the LATL in sentence processing in both modalities. These results thus show that the connectivity among fronto-temporal regions is upregulated for syntactic structure building in both sentence production and comprehension, providing further evidence for accounts of shared neural resources for sentence-level processing across modalities.

## INTRODUCTION

In moving away from the classical Wernicke-Lichtheim-Geschwind model of the neurobiology of language, it has become clear that the division of labour between fronto-temporal regions in processing language fundamentally rests on the connectivity between these and other brain regions (Dick et al., 2014; Hagoort, 2014; Hagoort & Beckmann, 2019). Much of the brain’s volume is constituted by white matter tracts that connect proximal and distal regions to one another. The advance of diffusion tensor imaging has enlarged the understanding of the orientation of white matter fibers and their terminations, adding to tracing and dissection studies in macaques and post-mortem brains (Rilling et al., 2008). In parallel, resting-state functional magnetic resonance imaging (fMRI) has advanced the understanding of large-scale networks independently of monosynaptic connections (Buckner et al., 2013). However, the effective modulation of networks by specific linguistic processing has only been studied sporadically, especially in language production, even though it has the potential to enable a deeper and broader understanding of how the coupling between brain regions is related to task-specific language production and comprehension (Friston, 2011; Hagoort & Beckmann, 2019).

The current understanding of the core white matter pathways that connect fronto-temporal regions centers on a dorsal and a ventral stream with separate functions (Dick et al., 2014; Friederici, 2012; Saur et al., 2008; Shekari & Nozari, 2023). The dorsal stream consists of the superior longitudinal fasciculus and the arcuate fasciculus, which have terminations in the posterior temporal lobe, inferior parietal cortex and posterior frontal cortex. These connections, especially the temporal projections, have been found to be enlarged in the human brain compared to the chimpanzee and macaque brains (Rilling et al., 2008; Sierpowska et al., 2022). These tracts have been suggested to have a role in phonological (Hickok & Poeppel, 2007) and syntactic processing (Friederici, 2012; Papoutsi et al., 2011; Zaccarella, Schell, & Friederici, 2017). The ventral stream consists of multiple fasciculi (uncinate fasciculus, extreme capsule, middle longitudinal fasciculus, inferior longitudinal fasciculus, and inferior fronto-occipital fasciculus), whose terminations and functions are still a matter of debate, but there is tentative consensus that they are involved in semantic processing (Dick et al., 2014; Friederici, 2012; Shekari & Nozari, 2023).

The functional connectivity between core language regions, which has been claimed to rely on the white-matter pathways introduced above (e.g., Friederici, 2011; Glasser & Rilling, 2008; Hagoort, 2014, 2017), has been investigated with functional neuroimaging studies that identify patterns of correlations between regions of interest (ROIs) and the rest of the brain (Biswal et al., 1995; Friston et al., 1995). Resting-state fMRI provides an overview of large-scale functional networks that are co-activated at rest, leading to a measure that is in-between anatomical connectivity and dynamic (transient) coupling (Buckner et al., 2013). At rest, the inferior frontal gyrus is functionally connected to parietal and temporal regions in a topographical organization that was suggested to indicate phonological, syntactic and semantic sub-networks across hemispheres, with the pars opercularis and the pars triangularis of the left inferior frontal gyrus (LIFGoper and LIFGtri) engaging the largest functional networks (Bulut, 2022; Przeździk et al., 2019; Xiang et al., 2010). Several regions in the temporal lobe also have wide co-activation patterns in resting-state fMRI, with the posterior middle temporal gyrus (pMTG) having the broadest connections to frontal and inferior parietal regions (Turken & Dronkers, 2011), resulting in a network that is highly similar to the regions usually found to be activated during language processing (e.g., Fedorenko et al., 2010; Giglio et al., 2022; Hagoort & Indefrey, 2014; Pallier et al., 2011; Segaert et al., 2012).

The functional coupling of networks in response to a task is instead studied with task-dependent or effective connectivity in task fMRI using, for example, psychophysiological interactions (PPI; Friston et al., 1997) or dynamic causal modelling (DCM; Friston et al., 2003). Such analyses model how the connectivity between frontal and temporal regions is modulated by the effect of a linguistic stimulus. For example, connectivity between LIFGoper and the left posterior temporal lobe (LPTL) was found to increase when resolving ambiguity in sentence comprehension (Snijders et al., 2010). Semantic composition has also been shown to drive increased coupling of LIFGtri, presupplementary motor area and posterior angular gyrus in comprehension (Graessner et al., 2021). Connectivity between language critical brain regions thus can be enhanced by specific linguistic processing during comprehension, but there is a lack of understanding of how the connectivity between fronto-temporal regions that is found in resting-state studies and comprehension task connectivity studies is regulated by language modality (i.e., production vs. comprehension). One study found that the LIFGoper was connected to a larger brain network in production than comprehension in a competition task involving object-relative clauses (Humphreys & Gennari, 2014), suggesting that functionally different networks may be engaged during production and comprehension.

Here, we asked how speaking versus listening affects the connectivity between fronto-temporal areas in a task manipulating syntactic structure building (Giglio et al., 2022). In this previous study, production and comprehension of the same stimuli engaged a similar network, but production elicited a stronger response to syntactic complexity, and frontal and temporal areas were engaged to a different extent in each modality. In particular, in production inferior frontal areas had a stronger response than in comprehension, while comprehension elicited stronger responses in the temporal lobe (Giglio et al., 2022). These regional differences across modalities could be due to production and comprehension relying on different functional sub-networks of the language system. Binder (2017) proposed a coherent temporal network engaged in sentence comprehension, centered in the pMTG for integration, that is supported by the extensive connectivity of the pMTG to other language-relevant regions relative to other temporal regions (Turken & Dronkers, 2011). This network could be functionally less engaged in production, as the final goal is to produce a motor output, after linearizing and articulating, which rely on frontal regions (Hickok & Poeppel, 2007; Kearney & Guenther, 2019; Matchin & Hickok, 2020).

Instead, the involvement of inferior frontal regions in production is generally agreed upon, with some debate on its specific function (Matchin & Hickok, 2020), but its role in comprehension is not settled. Proposals on the function of the LIFG in linguistic processing range from unification (independent of language modality; Hagoort, 2005, 2013) and phrase-structure building (unspecified for modality; Zaccarella, Meyer, et al., 2017; Zaccarella, Schell, & Friederici, 2017) to linearization (only in production; Matchin & Hickok, 2020) and working memory or top-down prediction in comprehension (Matchin et al., 2017; Matchin & Hickok, 2020). Coordination with inferior frontal regions for articulation and motor planning is nevertheless needed in production (Kearney & Guenther, 2019; Price, 2010), indicating a potentially stronger coupling between temporal and frontal regions during production than comprehension. It is less clear, instead, whether fronto-temporal coupling would be modulated differently by syntactic processing in each modality.

### The Current Study

In the current study, we directly addressed the question whether the connectivity of temporal and frontal regions previously found to be involved in linguistic processing differs between production and comprehension. To this end, we conducted a task-dependent connectivity analysis using generalized PPI (gPPI; McLaren et al., 2012) on a dataset that was previously collected to identify the linguistic network responsive to syntactic complexity in production and comprehension (Giglio et al., 2022). We focused on the connectivity of two seed ROIs, LIFGtri and LPTL, that were previously found to be involved in syntactic processing in both modalities (Giglio, Ostarek, Sharoh, et al., 2024; Matchin & Wood, 2020). We chose LIFGtri over LIFGoper, because LIFGoper was found to connect to other areas of the brain more broadly, which could suggest a lack of specificity for linguistic processing in its connectivity pattern and involvement in more general control and executive processes, while here we were primarily interested in characterizing the responses of a fronto-temporal network more specific for language (Bornkessel-Schlesewsky & Schlesewsky, 2013; Bulut, 2022; Humphreys & Gennari, 2014; see Fedorenko et al., 2012). We investigated the connectivity of seed regions LIFGtri and LPTL with two target ROIs, LIFGoper and left anterior temporal lobe (LATL), which were part of the constituent size effect in the previous study. To reduce individual variability, we focused on voxels that were responsive to constituent size in the previous study, and investigated how connectivity patterns from LIFGtri and LPTL to LATL and LIFGoper were affected by modality and syntactic complexity. We additionally tested for connectivity changes at the whole-brain level as a function of modality and complexity.

The current analysis expands on the previous one by identifying regions whose connectivity to the seed ROIs increases or decreases as a function of the task—here modality and constituent size. Functional connectivity as investigated with gPPI does not simply show which regions are coactivated but critically provides information on how connectivity between regions is driven by modality and syntactic complexity. If the differences in activation between frontal and temporal regions in production and comprehension found in the previous study (Giglio et al., 2022) were attributable to distinct networks for production and comprehension, we would expect a dissociation in the connectivity patterns across modality, with, for example, increased connectivity during syntactic processing between temporal regions in comprehension relative to production (Binder, 2017), and increased connectivity for syntactic processing between LIFG and LPTL in production compared to comprehension. If, instead, there was a single shared network for language that is modulated differently in each modality for auditory and motor functions, we would expect similar connectivity patterns for production and comprehension in the response to syntactic load.

## MATERIALS AND METHODS

### Participants

The analysis run in the current study was based on the data collected for a previous study (Giglio et al., 2022; Giglio, Ostarek, Weber, et al., 2024). Forty-six right-handed native Dutch speakers participated in the study after giving written informed consent (28 females, mean = 23.8 yr, range 19–35 yr). The study was approved by the ethical committee for human research for region Arnhem-Nijmegen. Participants reported having no language-related disorders and normal or corrected-to-normal vision and hearing. Six participants were excluded due to technical problems during preprocessing (*n* = 1), failing to complete the experiment leading to too little data (*n* = 2), and motion artefacts (*n* = 3). Forty participants remained for the analysis.

### Materials

The materials consisted of word sequences organized in three levels of constituent structure (C1, C2, C4), with 80 trials per condition. In C1, two verbs had to be produced in infinitival form, followed by two content words preceded by their determiner, leading to four constituents of one content word (C1: “klappen”, “slapen”, “de jongen”, “het meisje”, meaning “to clap”, “to sleep”, “the boy”, “the girl”). C2 consisted of two intransitive sentences composed of a subject and its verb, forming two constituents with two content words (C2: “de jongen slaapt, het meisje praat,” “the boy sleeps, the girl talks”). In C4, the second clause was embedded in the main clause with a complementizer phrase, leading to one sentence formed by four content words (C4: “de jongen hoort dat het meisje klapt,” “the boy hears that the girl claps”). The three conditions thus consisted of the same number of content words but differed in their constituent structure. The verbs were presented in root form (e.g., “klap,” “slaap”) and had to be inflected in all conditions. The verbs partly differed between conditions, since verbs allowing for a complementizer phrase (CP-verbs) are often not used in intransitive form. We selected 20 CP-verbs for the C4 condition, and repeated each four times in the C4 condition and four times in the C1 condition. We also selected 40 intransitive verbs, that were repeated four times in the C2 condition, twice in C4 and twice in C1. The CP-verbs and the intransitive verbs were matched in frequency (mean ± std: INT-verbs = 1.38 ± 0.88, CP-verbs = 1.46 ± 0.77, *t* = 0.59, *p* = 0.56) with SUBTLEX-NL (Keuleers et al., 2010), and concreteness (mean ± std: INT-verbs = 3.26 ± 0.67, CP-verbs = 3.21 ± 0.47, *t* = 0.27, *p* = 0.79; Brysbaert et al., 2014). There was also a filler condition to avoid too many verb repetitions, that used 80 transitive verbs used only once (“de man helpt de vrouw,” “the man helps the woman”). The nouns used in the sentences were always “the boy,” “the girl,” “the man,” “the woman” (“de jongen,” “het meisje,” “de man,” “de vrouw”).

**Figure 1.**
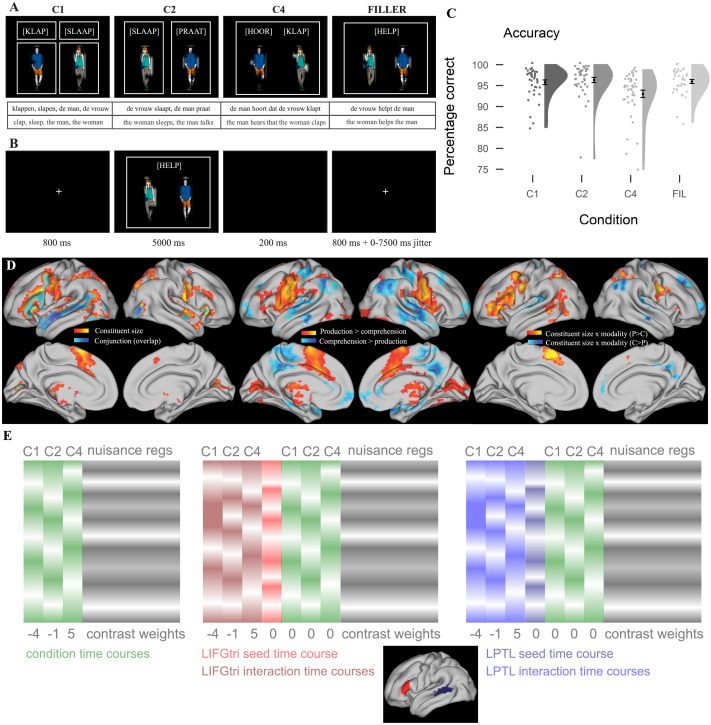
**A**. Visual stimuli for each of the conditions and its respective expected production output or comprehension input. **B**. Timing of each trial. **C**. Response accuracy in the production part of the task. **D**. Whole-brain results for the response to constituent size and modality and their interaction, as presented in Giglio et al. (2022). **E**. Design matrices for the different analyses. On the left, the design matrix for the activation analysis presented in the previous study (Giglio et al., 2022) is shown (relative to the activation shown in D). The middle and right matrices depict the design matrix used in the generalized psychophysiological interactions analysis, separately for each seed region. Since production and comprehension were run in separate blocks, the modality effects were across blocks. A, B, C, and D panels are adapted from Giglio et al. (2022). LIFGtri = pars triangularis of the left interior frontal gyrus, LPTL = left posterior temporal lobe.

The sentences were elicited by showing pictures for the nouns and written verbs (Figure 1A). The configuration of boxes around the verbs and pictures indicated the structure that had to be used by showing which elements should be combined in a sentence. In C1, four boxes indicated the production of four separate items (i.e., word sequences). In C2, two boxes surrounded one verb and one noun each, to form two sentences. In C4 and for fillers, one box surrounded all items to form one sentence that included all the items. Participants had no problems producing the correct output (mean accuracy was above 90% for all conditions, mean percentage correct: C1: 95.4, C2: 96.2, C4: 92.9, fillers: 95.9; Figure 1C).

### Experimental Procedure

First, there was a short behavioural practice session where participants read instructions for the task and practiced producing the sentences. The experimenter gave feedback to ensure correct understanding of the task. After the practice session, the fMRI experiment started. The experiment consisted of eight production runs interleaved by four comprehension runs, with a comprehension run always following two production runs. Each run included 40 trials and lasted approximately 5 min. Each trial began with a fixation cross for 800 ms, followed by a 5 s presentation of the picture screen, during which participants were instructed to produce or listen to the same materials (Figure 1B). The trial ended with a blank screen for 200 ms. Trial onset was jittered by 0–7,500 ms (mean 1,500 ms) based on design optimization for contrast effect detection (optseq2; Dale, 1999). In the comprehension runs, participants listened to recorded stimuli, which were presented 1 s after picture onset and lasted a maximum of 4 s (mean duration in s): C1 = 3.14, C2 = 2.46, C4 = 2.46, fillers = 1.79).

### fMRI Acquisition and Preprocessing

MR data were acquired in a 3T MAGNETOM PrismaFit MR scanner (Siemens AG, Healthcare Sector, Erlangen, Germany) using a 32-channel head coil. We collected a T1-weighted MRI scan for anatomical reference and several fMRI runs. The T1-weighted scan was acquired in the sagittal orientation using a 3D MPRAGE sequence with the following parameters: repetition time (TR)/inversion time (TI) 2,300/1,100 ms, echo time (TE) 3 ms, 8° flip angle, field of view (FOV) 256 mm × 216 mm × 176 mm and a 1 mm isotropic resolution. Parallel imaging (iPAT = 2) was used to accelerate the acquisition resulting in an acquisition time of 5 min and 21 s. Whole-brain functional images were acquired using a multiband (accelerator factor of 3) multiecho T2*-weighted sequence with the following parameters: TR 1,500 ms, TEs 13.4/34.8/56.2, flip angle 75°, FOV 84 mm x 84 mm x 64 mm, voxel size 2.5 mm isotropic. Fieldmap images were also acquired to correct for distortions along the phase-encoding axis. We acquired 12 fMRI runs per participant.

Preprocessing was performed using fMRIPrep 1.2.6-1 (Esteban, Blair, et al., 2018; Esteban, Markiewicz, et al., 2018). Each brain was spatially normalised to the ICBM 152 Nonlinear Asymmetrical template version 2009c. Susceptibility distortion correction was performed on all blood oxygen level dependent (BOLD) runs, which were slice-time corrected. Motion artifacts were estimated with ICA-AROMA (Pruim et al., 2015), whose noise regressors were later added to the first-level design matrix, together with head-motion parameters, framewise displacement, DVARS (Power et al., 2014), and anatomical component-based noise correction regressors (aCompCor; Behzadi et al., 2007).

### fMRI Connectivity Analysis

#### Seed ROI selection

Two seed regions were selected for the connectivity analysis. These were the pars triangularis of the LIFG and a region within the LPTL (see Figure 1E). The seed regions were selected functionally in each participant by extracting voxels that responded to constituent size individually in a LIFGtri and a LPTL mask. For the LIFGtri ROI, we used an anatomical mask of the LIFGtri based on the Harvard Oxford cortical atlas (Desikan et al., 2006). For the LPTL ROI, we used a mask based on the posterior temporal part of the conjunction effect of constituent size at the group level, since that region overlapped with the pMTG, the posterior superior temporal sulcus and the posterior superior temporal gyrus. We selected posterior voxels by selecting those that were posterior to Heschl’s gyrus, according to the Harvard Oxford cortical atlas. For both ROI masks, we then extracted voxels with *t* values > 2 at the subject level for the main effect of constituent size, thus focusing on voxels that were found to respond to constituent size independent of modality in each subject. This *t* threshold allowed us to have a substantial (>100) number of voxels in most participants for both ROIs (mean number of voxels ± std, LIFGtri: 167 ± 52; LPTL: 270 ± 82).

#### Whole-brain gPPI analysis

A gPPI analysis (McLaren et al., 2012) was conducted in AFNI (Version 22.1.09; Cox, 1996) to assess connectivity between regions as a function of constituent size and modality. We decided to use a gPPI analysis instead of the traditional PPI analysis, because gPPI is capable of modeling multiple experimental conditions in the same model. The procedure was as follows: first, we extracted the detrended time series for each seed ROI with AFNI programs 3dmaskave and 3dDetrend. The seed time series was then upsampled by a factor of 6 (0.25 s TR) and deconvolved with 3dTfitter to calculate the neuronal response. The interaction time series were then obtained by masking the neuronal response time course with a condition mask from each experimental condition. Condition masks had the value of 1 when a condition was present and 0 when a condition was absent. Interaction terms thus calculated for each condition were then convolved with the haemodynamic response function originally used in the first-level model, and then downsampled back to the original TR.

The interaction time series for each run, condition and modality and the seed time series were then included as additional regressors in the first-level design matrix (which was otherwise identical to the design matrix in Giglio et al. (2022), as reported below, Figure 1E). Two parallel analyses were run for the interaction time series extracted from each seed ROI (LIFGtri and LPTL). The preprocessed BOLD images in Montreal Neurological Institute standard space were smoothed with 4 mm full-width half-maximum Gaussian kernel in SPM12 (Version 7771; Penny et al., 2007) in Matlab2021a. The first-level design matrix included for each run as condition regressors the interaction time series of each condition and the seed timeseries of one ROI, correct trials for each of the four conditions, incorrect trials, the temporal derivative of each condition and parametric modulations of speech onset times (incorrect trials and parametric modulations were absent in the comprehension runs). Trial onset was picture onset time, and trial duration was the time from picture onset until speech offset. As nuisance regressors we included DVARS, framewise displacement, six aCompCor parameters, six motion parameters, and the AROMA noise components. Following parameter estimation, we extracted contrast images for each participant for the main effect of constituent size of the interaction regressors (with weights [−4 −1 5] based on the constituent size of the conditions), main effect of modality (production vs. comprehension), and the interaction between constituent size and modality. The contrast images were then tested at the group level with one-sample *t* tests following Henson (2015), with voxel-level threshold at *p* < 0.001 uncorrected, and a *p* < 0.05 family-wise error correction as a cluster threshold.

#### ROI gPPI analysis

In the ROI gPPI analysis, we assessed connectivity seeding from the LIFGtri and the LPTL and targeting the pars opercularis of the LIFG and the LATL, which were found to respond to constituent size in Giglio et al. (2022; Figure 1D). We also tested the connectivity between the seed ROIs. A similar procedure was used to identify the target ROIs as was used for the identification of the seed ROIs. First, we selected masks for the LIFGoper and the LATL. For LIFGoper, we used a mask of the pars opercularis based on the Harvard Oxford cortical atlas. For the LATL, we used the voxels anterior to Heschl’s gyrus in the conjunction effect of constituent size cluster in the temporal lobe at the group level based on Giglio et al. (2022). The ROIs were then restricted to include only those voxels that had a *t* value > 2 for the main effect of constituent size in each participant in each of these masks (mean number of voxels ± std, LIFGoper: 210 ± 57; LATL: 182 ± 60).

We then extracted mean beta values per participant for the interaction regressors for each condition and modality using MarsBar (Brett et al., 2002) in SPM12. We extracted the mean beta values in LIFGoper, LATL, and LPTL for the LIFGtri interaction regressors; and the mean beta values in LIFGoper, LIFGtri, and LATL for the LPTL interaction regressors. The beta weights were then compared with two separate mixed-effects models in R (Version 4.0.3) for each seed ROI using lme4 (Version 1.1-26; Bates et al., 2015). We used a linear contrast with weights [−4 −1 5] for constituent size. For modality, we used deviation coding. We used Helmert coding for the ROI factor, contrasting the ROIs by lobes and within lobes (i.e., for the LIFGtri model, we contrasted LIFGoper to the LATL and LPTL, and the two temporal regions to each other; for the LPTL model, we contrasted the LATL to the LIFG ROIs, and LIFGoper and LIFGtri to each other). We added by-participant random slopes for the interaction for ROI and modality and for the main effect of constituent size. We computed the contribution of factors using car (Version 3.1-0; Fox et al., 2021), and pairwise comparisons with emmeans (Version 1.7.5; Lenth et al., 2020).

## RESULTS

### ROI Results

#### LIFGtri seed ROI results

**Figure 2.**
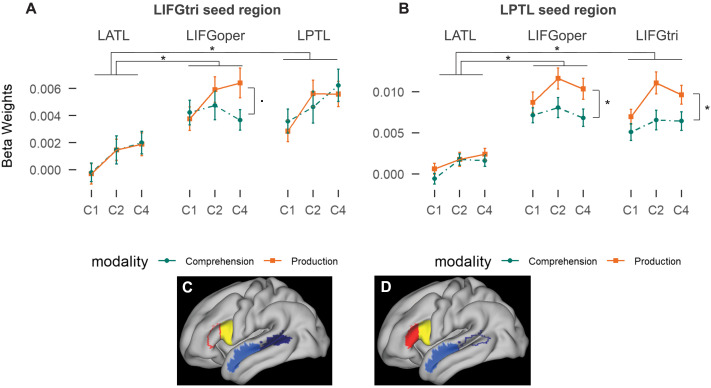
Average beta estimates of connectivity strength per condition, modality, and region of interest (ROI) from seed ROI LIFGtri in **A** and LPTL in **B**. **C** and **D** show the regions used for the ROI analysis, with the seed ROIs in outline. Red is LIFGtri, yellow is LIFGoper, dark blue is LPTL, light blue is LATL. Error bars represent standard error of the mean. * indicates *p* < 0.05, ▪ indicates *p* < 0.1. LIFGtri = pars triangularis of the LIFG, LATL = left anterior temperal lobe, LIFGoper = pars opercularis of the LIFG, LPTL = left posterior temporal lobe.

First, we focused on the connectivity between the two seed ROIs, LIFGtri and LPTL, and two target ROIs, LIFGoper and LATL (Figure 2). Note that because the analysis included two seed ROIs, *p* values below 0.025 are significant (Bonferroni corrected), but we report uncorrected *p* values for completeness. The model of the seed LIFGtri region connectivity with LATL, LIFGoper and LPTL indicated a significant main effect of ROI (*χ*^2^ = 56.4, *p* < 0.0001), of constituent size (*χ*^2^ = 13.6, *p* < 0.0003) and a trend for an interaction between modality, ROI, and constituent size (*p* = 0.072, *p* corrected = 0.146). Model outputs are reported in Table 1. Pairwise comparisons for the main effect of ROI indicated that the connectivity between LIFGtri and LATL was significantly lower than the connectivity of the LIFGtri with LIFGoper and LPTL (LIFGoper − LATL, LPTL − LATL: estimates > 0.0037, SE < 0.0007, *t* > 5.6, *p* < 0.0001). The effect of constituent size was present overall, but it was reduced with LIFGoper in comprehension, as suggested by the interaction. The slope for constituent size was marginally more positive in production than comprehension in LIFGoper (production − comprehension: estimate = 0.00034, *SE* = 0.00016, *t* = 2.08, *p* = 0.038, *p* corrected = 0.076), but did not differ in the other ROIs. Therefore, constituent size was related to an increase in the connectivity of the LIFGtri with all ROIs in both modalities. The connectivity between LIFGtri, LIFGoper, and LPTL was higher overall than between LIFGtri and LATL.

**Table 1.** Linear mixed model output for the connectivity of the LIFGtri seed with LIFGoper, LATL and LPTL as a function of constituent size, modality and ROI

Predictors	LIFGtri seed connectivity			
	Estimates	*SE*	Statistic	*p*
(Intercept)	0.003535	0.000406	8.702056	<0.001
Constituent size	0.000196	0.000053	3.688988	<0.001
Modality	−0.000158	0.000325	−0.486768	0.627
ROI1	0.001849	0.000251	7.368109	<0.001
ROI2	0.000629	0.000180	3.489866	0.001
Constituent size × Modality	−0.000051	0.000047	−1.087247	0.277
Constituent size × ROI1	0.000027	0.000058	0.472057	0.637
Constituent size × ROI2	−0.000052	0.000033	−1.551465	0.121
Modality × ROI1	0.000013	0.000244	0.053609	0.957
Modality × ROI2	−0.000208	0.000159	−1.307149	0.192
Constituent size × Modality × ROI1	0.000006	0.000058	0.106533	0.915
Constituent size × Modality × ROI2	−0.000060	0.000033	−1.800792	0.072

*Note*. Region of interest (ROI) was contrast coded with a Helmert contrast, where ROI1 contrasts LATL and LPTL, while ROI2 contrasts the two temporal regions with LIFGoper. LIFGtri = pars triangularis of the LIFG, LIFGoper = pars opercularis of the LIFG, LATL = left anterior temperal lobe, LPTL = left posterior temporal lobe.

#### LPTL seed ROI results

The model for the seed LPTL region connectivity with LATL, LIFGoper, and LIFGtri showed a similar pattern (Figure 2B and Table 2). There was a significant main effect of constituent size (*χ*^2^ = 6.9, *p* = 0.008), modality (*χ*^2^ = 6.8, *p* = 0.009), and ROI (*χ*^2^ = 86.9, *p* < 0.0001). Again, the connectivity of the LPTL with the LATL was significantly lower than with LIFGoper and LIFGtri (LIFGoper − LATL, LIFGtri − LATL: estimates > 0.0063, SE < 0.00083, *t* > 8.3, *p* < 0.0001). The connectivity with LIFGoper was also marginally larger than with LIFGtri (LIFGoper − LIFGtri: estimate > 0.0012, SE < 0.00048, *t* > 2.4, *p* < 0.055, *p* corrected = 0.11). There was also a marginally significant interaction between modality and ROI (*p* = 0.043, *p* corrected = 0.086). Pairwise comparisons indicated that connectivity was significantly higher in production than comprehension with LIFGoper and LIFGtri (production − comprehension: estimates > 0.0028, *SE* = 0.001, *t* > 2.73, *p* < 0.0095, *p* corrected < 0.019), but not with the LATL (production − comprehension: estimate = 0.0006, *SE* = 0.0007, *t* > 0.98, *p* = 0.33). Therefore, the connectivity of the LPTL with all ROIs increased with constituent size, and was higher with the LIFG ROIs than with the LATL. In addition, the connectivity between LPTL and LIFG ROIs was higher in production relative to comprehension.

**Table 2.** Linear mixed model output for the connectivity of the LPTL seed with LIFGoper, LIFGtri, and LATL as a function of constituent size, modality, and ROI

Predictors	Betas			
	Estimates	*SE*	Statistic	*p*
(Intercept)	0.005885	0.000465	12.647551	<0.001
Constituent size	0.000133	0.000051	2.635172	0.009
Modality	−0.001122	0.000341	−3.293730	0.001
ROI1	−0.000581	0.000240	−2.415515	0.016
ROI2	−0.002311	0.000249	−9.298264	<0.001
Constituent size × Modality	−0.000043	0.000051	−0.847982	0.397
Constituent size × ROI1	0.000069	0.000062	1.117230	0.264
Constituent size × ROI2	0.000031	0.000036	0.866759	0.386
Modality × ROI1	−0.000077	0.000244	−0.313094	0.754
Modality × ROI2	0.000396	0.000195	2.025742	0.043
Constituent size × Modality × ROI1	0.000022	0.000062	0.361089	0.718
Constituent size × Modality × ROI2	0.000027	0.000036	0.759535	0.448

*Note*. ROI was contrast coded with a Helmert contrast, where ROI1 contrasts LIFGoper and LIFGtri, while ROI2 contrasts the two frontal regions with LATL.

#### Constituent size

The ROI results thus indicate that the connectivity between and within frontal and temporal regions increased as a function of constituent size in both modalities. The constituent size effects observed especially in the LPTL connectivity did not always present as linear. Therefore, we explored whether a quadratic effect would better model the data. We ran the same models for the connectivity of the LPTL and the LIFGtri, but now the contrast for constituent size included both a linear and a quadratic term. The connectivity of the LIFGtri in response to constituent size was significantly linear (*β* = 0.0014, *SE* = 0.0003, *t* = 4.1, *p* = 0.0001) but not quadratic (*β* = 0.0005, *SE* = 0.0004, *t* = −1.5, *p* = 0.13). The connectivity of the LPTL in response to constituent size instead was significantly linear (*β* = 0.0011, *SE* = 0.0004, *t* = 2.98, *p* = 0.0048) as well as quadratic (*β* = 0.0013, *SE* = 0.00005, *t* = −2.6, *p* = 0.012), but neither contrast interacted with modality or ROI. Therefore, although the effect was not consistently linear, it was also not reliably quadratic in the connectivity with any ROI, suggesting that the quadratic effect was not modality or region specific.

Overall, the results showed that the LIFG and the LPTL form a stronger network that seems to connect with the LATL only for the sentence conditions (C2 and C4). Finally, the connectivity within the temporal lobe was not affected by modality, but the connectivity between LPTL and frontal regions, and less robustly within frontal regions, was increased during production. It should be noted that the increased connectivity between LPTL and LIFG in production was only found with LPTL as seed region, but not when LIFGtri was seed region as noted above. As gPPI does not allow for directed inferences, it is presently unclear what significance this result has for the description of this network. For example, the results might be explained by a network configuration where LIFGoper functions as an intermediate region between LPTL and LIFGtri, but potential explanations for this asymmetry cannot be adjudicated by the current experiment or analysis.

### Whole-Brain Results

**Figure 3.**
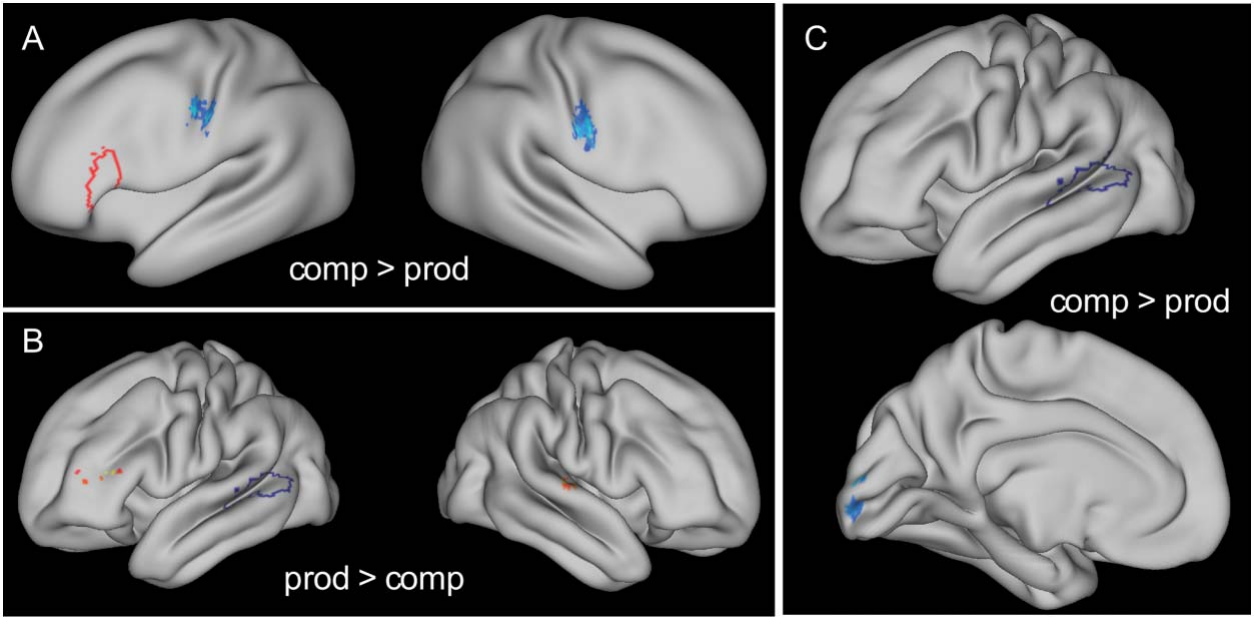
Whole-brain results for the main effect modality from seed LIFGtri in **A** (on inflated brain to show extent of clusters) and seed LPTL in **B** and **C**. Seed regions are shown in outline in red and blue, while significant clusters (thresholded at *p* < 0.05 family-wise error-corrected) are colored in warm colors for production > comprehension effects, and in cold colors for comprehension > production effects.

In addition to investigating the connectivity between regions in the frontal and temporal lobes, we investigated the connectivity of the LIFGtri and the LPTL with other areas of the brain as a function of modality and constituent size. We found a main effect of modality for the LIFGtri seed, showing increased connectivity in comprehension over production to the left and right precentral to postcentral gyri (Figure 3A and Table 3). The LPTL seed instead was more strongly connected to the posterior occipital cortex in comprehension than in production (Figure 3C). These comprehension effects seem to suggest task-related effects, possibly related to visual processing of the pictures.

**Table 3.** fMRI whole-brain summary of cluster peak coordinates and statistics

**Seed ROI**	**Contrast**	**Cluster**	Size	**Peak voxel (MNI coordinates)**				**Peak anatomical location**
		*p* (FWE-corrected)		Max *t* value	*x*	*y*	*z*	
LIFGtri	Comprehension > Production	0	83	4.67	54	−7	30	R postcentral gyrus (extending into precentral)
		0	64	4.62	−48	−10	37	L precentral gyrus (extending into postcentral)
LPTL	Comprehension > Production	0	55	5.07	−6	−100	12	L occipital pole
LPTL	Production > Comprehension	0.006	37	5.49	−44	50	12	L anterior middle frontal gyrus
		0.006	37	4.61	62	−12	7	R superior temporal gyrus
		0.035	27	4.58	−28	8	62	L posterior middle frontal gyrus

*Note*. *P* values below 0.025 are significant after correcting for multiple tests (from two seed ROIs), but all peaks are shown for completeness.

The LPTL also had increased connectivity to a small cluster in the LIFGtri in production compared to comprehension, indicating that the temporal-frontal connectivity was upregulated during production, as already indicated by the ROI results (Figure 3B). There was also increased connectivity to a right-lateralised cluster in the superior temporal cortex, possibly indicating monitoring needs in production (Kearney & Guenther, 2019). There were no significant clusters for the main effect of constituent size or for the interaction between constituent size and modality. Since the ROI results for the most part did not appear in the whole-brain results, we present uncorrected results with a higher threshold in Figures S1–S4 in the Supporting Information, available at https://doi.org/10.1161/nol_a_00154, which also includes clusters in the ROIs analysed here.

## DISCUSSION

We analysed task-based functional connectivity among regions of the fronto-temporal language network to obtain further insights into the coupling of regions involved in linguistic processing as a function of language modality. We investigated the connectivity of seed region LIFGtri with target regions LIFGoper and two temporal clusters, and to the whole-brain. We also investigated the connectivity of the left posterior temporal cluster with the anterior temporal cluster and the two LIFG regions. All of these regions were previously found to be modulated by constituent size during production as well as comprehension (Giglio et al., 2022). We found that the functional connectivity among these regions was enhanced for larger syntactic constituents. In addition, the connectivity between the posterior temporal lobe and the inferior frontal gyrus was enhanced in production relative to comprehension. The anterior temporal cluster, while showing enhanced connectivity to LPTL and LIFGtri as a function of constituent size, was relatively less coupled to the LPTL and LIFGtri than these latter regions were connected to each other.

The connectivity between the LPTL and the LIFG was (i) upregulated with larger constituent size, (ii) upregulated in production over comprehension, and (iii) greater than the connectivity between either region and the LATL. This result highlights a dorsal network that is functionally connected in both modalities as a function of constituent structure building. The nature of the present study does not allow us to be certain about the white matter tracts underlying the functional connectivity. Studies of structural connectivity suggest that these regions are connected via the dorsal stream through the arcuate fasciculus (Dick et al., 2014; Friederici, 2012), although the connectivity between the LPTL and the LIFGtri could also be supported by ventral stream fasciculi. Previous studies, however, highlight the importance of the dorsal stream for syntactic processing. The arcuate fasciculus is found to mature in the developing brain in relation with syntactic abilities (Skeide et al., 2016). In addition, the functional coupling between LIFG and LPTL has been found to be critical for syntactic and compositional processing (den Ouden et al., 2012; Griffiths et al., 2013; Wilson et al., 2012). When disrupted in patients, it was seen to lead to syntactic impairments (Papoutsi et al., 2011). The current results thus show that the functional network for syntactic processing relies on the connectivity between frontal and temporal regions and is common between production and comprehension.

Our results are agnostic as to the origin and direction of the connectivity between these areas. One study that used dynamic causal modelling to generate predictions about the directionality of connections in comprehension found evidence for a model where the LIFG was the driving input in the connectivity with the LPTL, and syntactic complexity modulated the connectivity in this direction (den Ouden et al., 2012). Another fMRI study instead found that sentence complexity in comprehension drove an increase in the LPTL earlier than in the LIFG (Uddén et al., 2022). In magnetoencephalography (MEG), the connectivity between fronto-temporal regions has been found to be driven by outflow from middle temporal regions, with frontal regions receiving input and sending output to other frontal regions (Hultén et al., 2019; Schoffelen et al., 2017). Another MEG study found that in verb-object compositionality the LMTG affected responses in LIFG, which in turn connected back to LMTG (Lyu et al., 2019), highlighting bidirectional connectivity. These findings thus are consistent with the hypothesis that the LPTL and LIFG interact in a dynamically reverberating network that supports the integration of memory and unification components of language processing following the Memory, Unification and Control (MUC) model (Baggio & Hagoort, 2011; Hagoort, 2013). Our results, therefore, add to the growing literature on the interconnectivity of frontal and temporal regions by extending the evidence to sentence production.

As the current gPPI analysis does not allow for directionality inferences, it is left open whether modality would affect the directionality of interactions. Production and comprehension are often discussed as having different input/outputs, which could suggest differences in the timing of recruitment of different regions and perhaps of the directionality of connectivity between regions (Gambi & Pickering, 2017; Pickering & Garrod, 2013). Giglio, Ostarek, Sharoh, et al. (2024), for example, found that the PTL responded to syntactic structure building later in production but earlier in comprehension. However, even hypothesizing differences in the interaction dynamics between modalities, they may nevertheless happen at timescales too short to be detectable in fMRI if the network is as dynamically reverberating as suggested by Lyu et al. (2019). Within the aims of the current study, it is clear not only that production and comprehension recruit the same network (Arvidsson et al., 2024; Giglio et al., 2022; Hu et al., 2023; Menenti et al., 2011; Menenti et al., 2012; Segaert et al., 2012), but also that building syntactic structure relies on the connectivity between regions of this network in a similar way in production and comprehension. Even though production was seen to elicit a stronger response in the LIFG, and comprehension a stronger response in the LMTG (Giglio et al., 2022), production and comprehension both rely on these regions and, critically, on their similar coupling for higher linguistic processing. This shared network modulation provides the neural basis for theories that propose shared representations and processes in production and comprehension (Humphreys et al., 2016; Kempen, 2000; Momma & Phillips, 2018; cf. Meyer et al., 2016).

We also found a production-specific effect, which we argue is due to production-specific requirements for articulation. The connectivity between the posterior temporal lobe and the IFG was increased in production relative to comprehension. Interestingly, this modality effect complements the modality asymmetry found in Giglio et al. (2022), where the LIFG was more active in production than comprehension, while the LPTL was more active in comprehension. The modality activation imbalance is potentially related to the modality-affected coupling between posterior temporal and inferior frontal regions. In fact, the increased connectivity in production may upregulate the engagement of the LIFG. It is important to note that the lack of an interaction between modality and constituent size (which was instead found in the activation results; Giglio et al., 2022) indicates that the modality effect in LIFG-LPTL connectivity was not related to the complexity of syntactic processing. Rather, it may reflect production-specific computations, such as coordination of sequences for phonetic encoding and articulation (Hickok & Poeppel, 2007). Sentence production ends with a motor output that is coordinated by premotor regions in the frontal cortex and may rely on inferior frontal regions for linearization, while comprehension, especially in the current design, does not culminate in an action (Kearney & Guenther, 2019; Matchin & Hickok, 2020; Ries et al., 2019). Alternatively, the increased connectivity with the LIFG in production may be due to increased executive or working memory demands in production, which have been hypothesized to be related to LIFG activity (Acheson & MacDonald, 2009; Matchin, 2018; Rogalsky et al., 2008), since production is in itself a “task,” while comprehension, as manipulated in the current study, was a more passive activity.

This functional production network seems to further rely on the enhanced connectivity between LIFGtri and LIFGoper in the production of larger constituents, possibly suggesting the stronger need to coordinate a sentence-level linked articulatory plan (Basilakos et al., 2018; Flinker et al., 2015; Hickok & Poeppel, 2007). Moreover, the stronger connectivity between inferior frontal regions for larger constituents may derive from language production not being able to rely on good-enough processing. In contrast, sentence comprehension can be successful with only partial structural analysis (Ferreira, 2003; Ferreira et al., 2002; Goldberg & Ferreira, 2022). However, the similar increase in connectivity between LPTL and LIFG for constituent size in production and comprehension would seem to suggest that both of these regions are engaged during syntactic processing in comprehension, contrary to theories that see LIFG involvement as only necessary for linearization in production (Matchin & Hickok, 2020).

It was remarkable that, independently of modality, the LATL was less coupled to the LPTL-LIFG network, while being similarly sensitive to constituent size as the other regions. This region is connected to the LIFG via the ventral stream, thought to be involved in semantic processing, distinct from the dorsal stream involved in phonological and syntactic processing (Dick et al., 2014; Friederici, 2012; Hickok & Poeppel, 2007). The reduced connectivity with adjacent posterior temporal regions may thus reflect its being part of not only a different anatomical but also a functional network. However, its connectivity was enhanced for larger constituents in this region, which suggests that it was nevertheless involved in the large network involved in language processing in this experiment (that also included parietal and middle frontal regions). The increased response and connectivity of the LATL cluster for larger constituents could be due to its involvement in the access of phrase-structure templates (Friederici, 2012), or in larger semantic composition needs for larger sentences (Bornkessel-Schlesewsky & Schlesewsky, 2013; Hickok & Poeppel, 2007; Matchin & Hickok, 2020; Pylkkänen, 2020). Pallier et al. (2011) also showed that activity in the LATL increased with constituent size, but only for semantically meaningful sentences, relative to jabberwocky sentences, suggesting its involvement in semantic composition effects.

The constituent size effect, though reliable in the ROI analysis, did not surface at the whole-brain level. Inspection of the slopes for the response to constituent size suggests that the lack of change in connectivity at the whole-brain level could be due to the effect not always being linear. The finding of a quadratic as well as a linear response to constituent size in the connectivity seeding from the LPTL supports this interpretation. In fact, in some ROIs the constituent size effect had a decreasing trend after C2, suggesting that rather than being sensitive to constituent size itself, the connectivity may be enhanced with sentences (C2 and C4) relative to word lists (C1), again indicating that functional connectivity increases with the interaction of memory and unification (Hagoort, 2014), but not necessarily as a function of increasing syntactic complexity. Since the shape of the connectivity in response to constituent size was not reliably different between ROIs, it is difficult to say whether the linear and quadratic effects reflect stable features of the connectivity between fronto-temporal regions.

The whole-brain results for modality were partly surprising, identifying a network for comprehension that was not expected a priori. In fact, the increased connectivity in comprehension of the LIFGtri with a bilateral cluster bordering the central sulcus, and the connectivity of the LPTL with an occipital cluster seemed to be related to task-specific effects rather than higher-level linguistic processing. Both of these clusters were found to respond more to production than comprehension in the previous study. This was a production vs. comprehension contrast rather than vs. baseline, so an increase in connectivity in comprehension corresponds to a decrease in connectivity in production. It is possible that in both cases in production there was a more complex network, with an intermediate region which reduced the direct connectivity between these regions. An intracranial electroencephalography study on object naming found negative correlations between LIFGtri and a region of the motor cortex prior to articulation, while there were positive correlations with LIFGoper (Conner et al., 2019). The more lenient whole-brain results (see the Supplementary Information) identified increased connectivity between the LIFGtri and a cluster in the middle frontal cortex adjacent to precentral cortex in production, which could have been the intermediate region supporting articulation and decreasing the direct connectivity between LIFGtri and the precentral gyrus. This negative correlation in production may have then manifested as a positive interaction during comprehension. Similarly, the increased temporo-occipital connectivity in comprehension found in the whole-brain analysis might be due to there being sustained attention on the visual input during comprehension compared to production, because of lower-level task demands, such as matching auditory input with visual input. This direct connectivity may have been reduced in production again due to an intermediate region, such as the ventro-occipitotemporal cortex, reducing the connectivity between these two more distal regions. To process the verb roots, visual and linguistic information may have been integrated in the ventro-occipitotemporal cortex for reading in production (Sharoh et al., 2019), but not in comprehension where reading was not necessary for the task.

In summary, we found a dynamic posterior temporal–inferior frontal network to be upregulated for syntactic structure building in both production and comprehension, suggesting similar neural resources for sentence processing in both modalities and reiterating the importance of the dorsal stream for syntactic processing. This posterior temporal–inferior frontal connectivity was upregulated in production due to production-specific linearization and articulatory needs. A mid-anterior temporal cluster was also upregulated for constituent structure building but it was functionally less co-activated with the posterior temporal lobe and the IFG, suggesting that the ATL is part of a different functional network for language processing, that can be flexibly recruited to support sentence-level processing. Overall, the connectivity results highlight that a shared brain network is upregulated for linguistic processing similarly in production and comprehension, with differences in connectivity patterns likely due to modality-specific demands.

## ACKNOWLEDGMENTS

This work was supported by the Max Planck Society. We would like to thank Eva Poort for help with stimulus recording and coding of production recordings and Maarten van den Heuvel for help with presentation scripts.

## FUNDING INFORMATION

Peter Hagoort, Max-Planck-Gesellschaft (https://dx.doi.org/10.13039/501100004189). Peter Hagoort, Nederlandse Organisatie voor Wetenschappelijk Onderzoek (https://dx.doi.org/10.13039/501100003246), Award ID: 024.001.006.

## AUTHOR CONTRIBUTIONS

**Laura Giglio**: Conceptualization: Lead; Data curation: Lead; Formal analysis: Lead; Investigation: Lead; Methodology: Equal; Project administration: Lead; Visualization: Lead; Writing – original draft: Lead. **Daniel Sharoh**: Conceptualization: Equal; Methodology: Equal; Software: Lead; Writing – review & editing: Equal. **Markus Ostarek**: Conceptualization: Equal; Supervision: Equal; Writing – review & editing: Equal. **Peter Hagoort**: Funding acquisition: Lead; Resources: Lead; Supervision: Lead; Writing – review & editing: Equal.

## DATA AND CODE AVAILABILITY STATEMENT

The raw data, including imaging data and stimuli, are available on the Radboud Data Repository (Giglio, Ostarek, Weber, et al., 2024) at https://doi.org/10.34973/xnhy-md07. The functional connectivity data and code are available on the Radboud Data Repository (Giglio, Sharoh, et al., 2024) at https://doi.org/10.34973/2b5y-6c45.

## Supplementary Material



## TECHNICAL TERMS

Functional connectivity: As used in this study, reveals whether the interaction between two brain regions is affected by a task.
gPPI: Allows for modeling all conditions in an experiment, relative to traditional psychophysiological interactions analyses that contrast only two conditions.
