# Supplementary material for: Connectivity of Fronto-Temporal Regions in Syntactic Structure Building During Speaking and Listening
Source: Neurobiol Lang (Camb). 2024 Oct 8;5(4):922–41. doi: 10.1162/nol_a_00154 (PMC11495677; doi:10.1162/nol_a_00154)
Supplement: Supplementary file 1 [file nol-5-4-922-s001.pdf]

Supplementary Information

The whole-brain gPPI results appeared to be underpowered relative to the ROI analysis. To check for consistency of whole-brain responses with the ROI analyses, we report whole-brain results using a more lenient threshold ($p < 0.05$ uncorrected) for the main effects of constituent size and modality and for the interaction between constituent size and modality, separately for LIFGtri seed (Fig. S1-2) and for LPTL seed (Fig. S3-4). These uncorrected responses include clusters in the target ROIs as well as a few additional areas that were also found in the activation results (Fig. 1D).

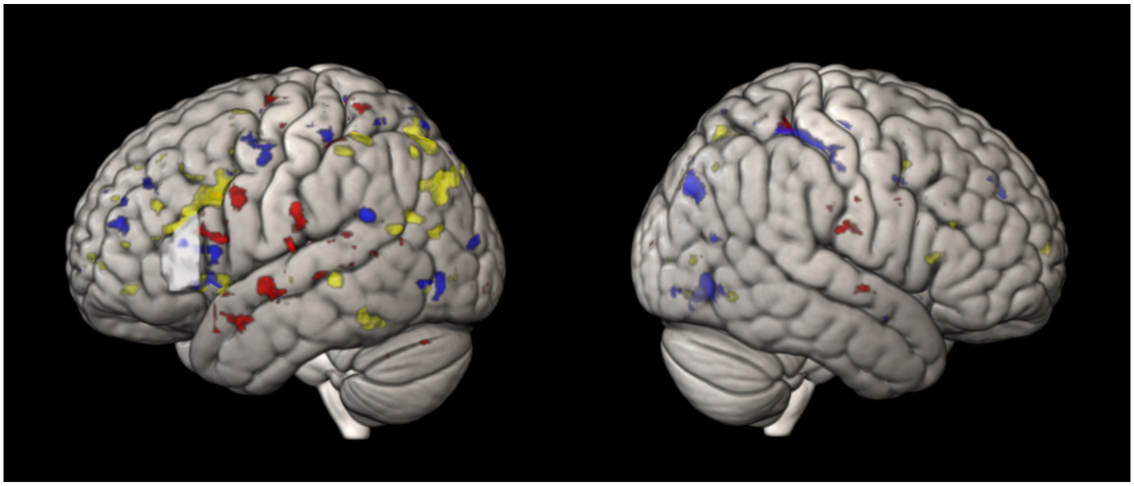

Supplementary Figure 1: *Whole-brain increase in connectivity as a function of constituent size (red), modality (production>comprehension, yellow), and the interaction between modality and constituent size (production>comprehension, blue). The seed ROI (LIFGtri) is shown in white. The results are thresholded at p < 0.05 uncorrected.*

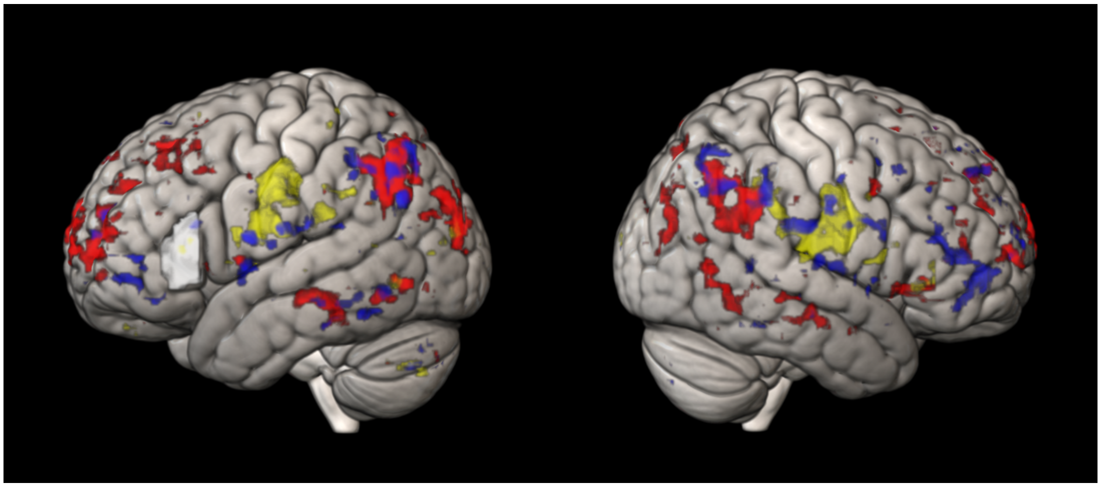

Supplementary Figure 2: *Whole-brain connectivity as a function of a decrease in constituent size (red), modality (comprehension>production, yellow), and the interaction between modality and constituent size*

(comprehension>production, blue). The seed ROI (LIFGtri) is shown in white. The results are thresholded at $p < 0.05$ uncorrected.

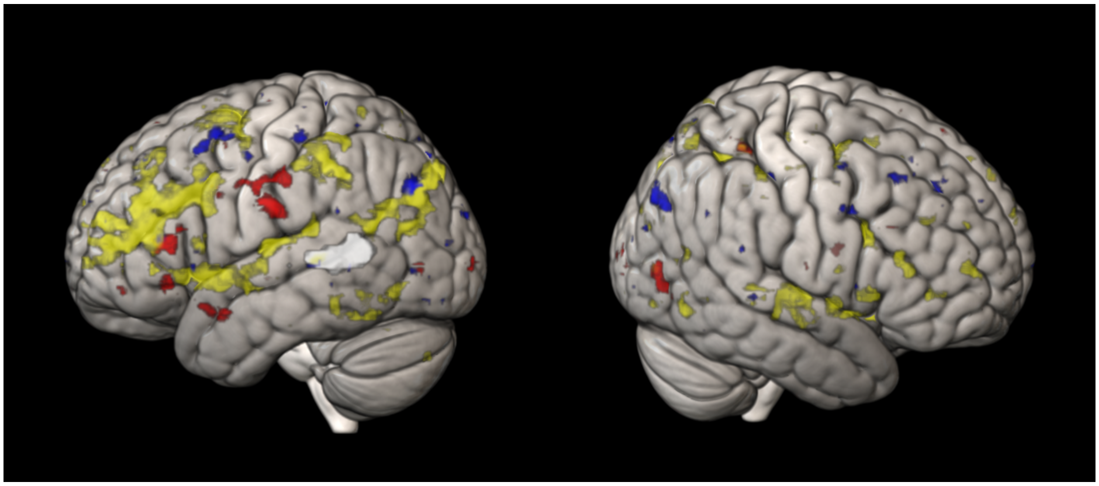

Supplementary Figure 3: *Whole-brain increase in connectivity as a function of constituent size (red), modality (production>comprehension, yellow), and the interaction between modality and constituent size (production>comprehension, blue). The seed ROI (LPTL) is shown in white. The results are thresholded at $p < 0.05$ uncorrected.*

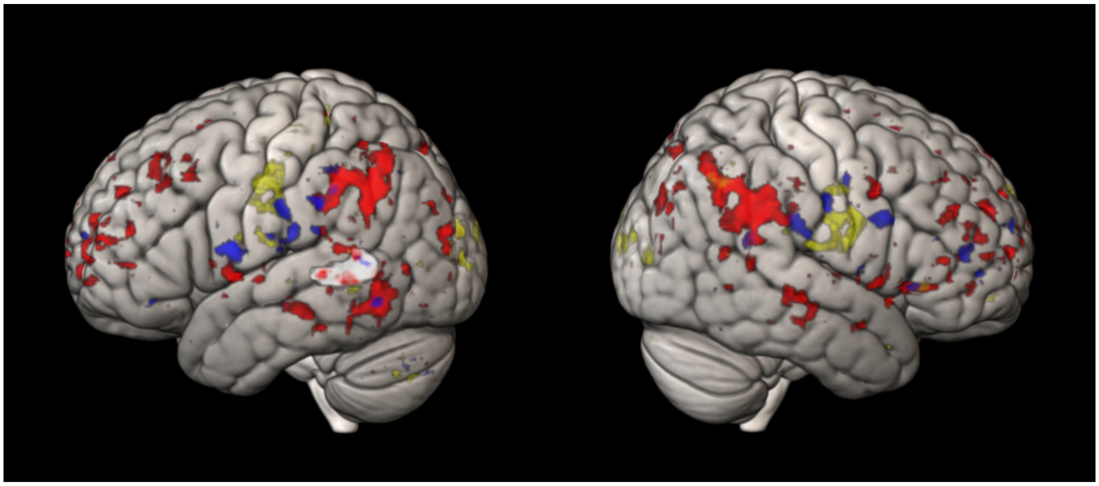

Supplementary Figure 4: *Whole-brain connectivity as a function of a decrease in constituent size (red), modality (comprehension>production, yellow), and the interaction between modality and constituent size (comprehension>production, blue). The seed ROI (LPTL) is shown in white. The results are thresholded at $p < 0.05$ uncorrected.*
